# Department of Practice of Medicine

**Published:** 1894-09

**Authors:** 

**Affiliations:** Galveston, Professor of Pathology and Lecturer on Mental and Nervous Diseases in the Medical Department of the University of Texas, Galveston


### Current Medical Literature.

### DEPARTMENT OF PRACTICE OF MEDICINE.

EDITED BY PROF. ALLEN J. SMITH, M. D., GALVESTON,
Professor of Pathology and Lecturer on Mental and Nervous Diseases in the Medical Department of the University of Texas, Galveston.

SO-CALLED SPONTANEOUS COMBUSTION.—Dr. Adrian Hava, of New Orleans (*New Orleans Med. and Surg. Journal*, April, 1894), relates of his father, Dr. John Joseph Hava, a practitioner of medicine in the island of Cuba, that in 1862 he was called to a plantation to see an old negress, a slave of the plantation, whom he had treated for some form of paralysis. This woman was very corpulent, and had been almost constantly occupied for more than twenty years in parching coffee for the daily use of the plantation. She always seemed to be partly under the influence of liquor, never perspiring, and even in the warmest weather was always complaining of feeling cold, and was always to be found around a furnace. One morning she was discovered dead and burning, and Dr. Hava arrived in time to witness the fact. The room was filled with smoke, and the odor coming from the partly consumed body was characteristic of burning human flesh. The parts charred and yet in combustion were the thighs, abdomen and chest, the end of the limbs and head being spared, and none of the combustible objects in the room had been burned. The case was reported at the time to the Academy of Sciences of Havana.

Having become thus persuaded by his father's experience of the fact that the human body may become under some unknown circumstances of a combustible nature, Dr. Adrian Hava in 1880 undertook a number of investigations which he has pursued until the present with a view of discovering the explanation of the phenomena in this and other similar cases reported from time to time. In studying the records of most recorded cases it is to be noted that alcoholic abuse has been suspected and theoretically held as causative. Most cases have occurred in cold climates or cold seasons; the subjects were fat, usually fat females, sluggish, drowsy, generally suffering from a chronic bronchitis, always complaining of feeling cold and living in some small, poorly ventilated room in which was a furnance or foot-warming brazier burning charcoal. As in no instance had the victim been known to call for help, and as the hands exhibited no signs of burns as from efforts to overcome the combustion, it seems reasonable to believe that either life was extinct, or that at least there was unconsciousness when the burning took place. The inflamed blistered appearance of the skin near the burned parts suggest that the burning took place either in life or just after death. According to records the combustion was always a rapid one, the body burning with a pale, flickering or lambent flame, increased by the sprinkling of water upon it. The carbonized flesh retained its appearance in shape, but was porous and readily crumbled under the touch; and the unconsumed parts were of a bright red hue. Under and about the body was to be found melted human fat, said to burn readily if ignited. Ordinary efforts to burn it have abundantly proved that the human body is not combustible, and will not continue to burn on its own account, even if its vessels be injected with alcohol, and it has been steeped for a long time in this fluid, the flames in such cases dying out as soon as the alcohol on the surface has been consumed. It has been stated as sustaining the view that alcohol is causative of this condition of the body, that in a few instances where it was sought alcohol has been found in the blood and tissues of drunkards who had died after imbibing considerable amounts of alcoholic fluid; but other than this fact and the suspicion of alcoholic abuse in case of the subjects of the combustion there is no proof of its causative relation. In order to satisfy himself upon this point the author administered to a series of a dozen young roosters half a drachm or more of brandy three times a day for more than a year —the cocks being kept intoxicated for from 4 to 16 hours daily. At the expiration of fourteen months but two of the fowls remained alive, the other ten having all died at some phase of the experiment, search for alcohol in their tissues in every instance having been negative. Six more cocks were added, but at the end of two more months the two remaining from the original lot were dead as well as four of the six last procured; and no alcohol had been found. Of the remaining two, one morning immediately after the usual dose of brandy had been administered, one was accidentally killed by a horse; and examination of the tissues of the stomach some hours later showed the abundant presence of alcohol. This instance and others suggested by it led to the conclusion that alcohol can only be found in the blood and tissues of individuals dead shortly after having imbibed an alcoholic fluid, any alcohol ingested some time before death being converted into $CO_2$ and water. Having thus satisfied himself that alcohol could not be retained in the body in sufficient amount to render the latter inflammable, the author turned his attention to the consideration of CO gas, remembering the known effects of the gas which is given off in large amount from heating apparatus such as was so constantly found in connection with these cases of "spontaneous combustion," miscibility with air, its manner of burning with a pale blue, lambent flame, it production of headache, dizziness and nausea when first inhaled, the ready replacement of these first symptoms by feelings of prostration, chilliness, bronchial irritation, redness of the face and skin, an irresistible desire to sleep, prolonged insensibility and eventually death, and, too, the occasional production of various forms of paralysis after recovery from the coma. The author deemed that it was possible that this gas might be accumulated in the system, perhaps in its combination with hæmoglobin, to such an extent as to give rise to the phenomena of combustion if opportunity for ignition were afforded. Therefore in a glass case, a number of rabbits were placed from four to six times daily, and CO gas introduced slowly until they became more or less insensible, the animals being withdrawn each time to fresh air to recover their senses. Their appetites seemed rarely impaired and the animals were given as much food as they would take. These experiments were kept up several years, some animals dying from time to time and being replaced by others. The result was successful, the tissues of animals thus subjected to carbon monoxide gas becoming in the end inflammable. The shortest time in which sufficient carbon monoxide was accumulated in a rabbit's tissues so that combustibility resulted was 169 days of continued and careful administration of the gas. The skin and subcutaneous tissues as well as the muscular tissues were readily combustible, burning with a bluish flame, leaving porous carbonized masses retaining the shapes of the parts that had been consumed, but producing very little smoke because of the absence of fat. This carbon monoxide is held in the diffused hæmoglobin in the muscle tissue. The "spontaneous combustion" of human bodies is the result which may possibly take place after gradual, constant and progressive accumulation of the gas for years, provided the accumulation is great enough, and provided further, that the body find itself in contact with a flame or heat sufficient to cause ignition. These condi tions being fulfilled, as might accidentally take place when the individual has been overcome by the gas and fallen over the "*chauffante*" containing burning coals, the CO begins to burn, producing an intense heat and melting the fat and carbonizing the skin, subcutaneous and muscle tissue; this carbonized tissue being very porous acts as does the wick in a lamp and absorbs the melted fat. Then the fat burns, and as long as there is fat present to be melted by the heat of the burning CO gas the body continues to burn. The abdominal tissues burn most readily, not only because there occurs in this region an especial amount of fat, but because also there is found here an extra deposit of the carbon monoxide, from the collection in the abdominal viscera of blood loaded with the combination of the gas and the hæmoglobin. This alteration of the blood accounts for the inabability to dispose of the fat brought into the system and its consequent accumulation, and also for the sensations of cold so commonly experienced.

These experiments of Dr. Hava are of great importance and have resulted in one of the most interesting discoveries of recent years. Moreover they open a new avenue for medico-legal thought, it being possible in the light thus brought upon certain cases held as the results of efforts of homicides to destroy the body of a victim by burning, that an altogether innocent being may be accused of a deed which was never committed but occurred according to this law of nature, hitherto unknown.

